# Identification and Characterization of Porcine Kobuvirus Variant Isolated from Suckling Piglet in Gansu Province, China

**DOI:** 10.3390/v5102548

**Published:** 2013-10-18

**Authors:** Shengtao Fan, Heting Sun, Ying Ying, Xiaolong Gao, Zheng Wang, Yicong Yu, Yuanguo Li, Tiecheng Wang, Zhijun Yu, Songtao Yang, Yongkun Zhao, Chuan Qin, Yuwei Gao, Xianzhu Xia

**Affiliations:** 1 Institute of Laboratory Animal Sciences, Chinese Academy of Medical Sciences & Peking Union Medical College, Beijing 100021, China; E-Mails: fst0007@163.com (S.F.); zhijun0215@gmail.com (Z.Y.); chuanqin@vip.sina.com (C.Q.); 2 Key Laboratory of Jilin Province for Zoonosis Prevention and Control, Institute of Military Veterinary, Academy of Military Medical Sciences, Changchun 130122, China; E-Mails: xiaofengsht@163.com (H.S.); gaoxiaolong8905@163.com (X.G.); liyuanguo0520@163.com (Y.L.); wgcha@163.com (T.W.); yst610223@hotmail.com (S.Y.); zhaoyongkun1976@126.com (Y.Z.); 3 College of Animal Science and Technology, Jilin Agricultural University, Changchun 130118, China; E-Mails: blueinfantastic@126.com (Y.Y.); xiaoyaojingtina@126.com (Z.W.); jordanyuyicong@163.com (Y.Y.)

**Keywords:** porcine kobuvirus, variation, phylogenetic analysis

## Abstract

Kobuviruses comprise three species, the Aichivirus A, Aichivirus B, and Aichivirus C (porcine kobuvirus). Porcine kobuvirus is endemic to pig farms and is not restricted geographically but, rather, is distributed worldwide. The complete genomic sequences of four porcine kobuvirus strains isolated during a diarrhea outbreak in piglets in the Gansu province of China were determined. Two of these strains exhibited variations relative to the traditional strains. The potential 3C/3D cleavage sites of the variant strains were Q/C, which differed from the Q/S in the traditional porcine kobuvirus genome. A 90-nucleotide deletion in the 2B protein and a single nucleotide insertion in the 3′UTR were found in the variant strains. The VP1 regions of all four porcine kobuviruses in our study were highly variable (81%–86%). Ten common amino acid mutations were found specifically at certain positions within the VP1 region. Significant recombination sites were identified using SimPlot scans of whole genome sequences. Porcine kobuviruses were also detected in pig serum, indicating that the virus can escape the gastrointestinal tract and travel to the circulatory system. These findings suggest that mutations and recombination events may have contributed to the high level of genetic diversity of porcine kobuviruses and serve as a driving force in its evolution.

## 1. Introduction

Picornaviruses (Picornaviridae family) are small, non-enveloped viruses with single-stranded, positive-sense genomic RNA. This family is divided into the following 17 genera: Aphthovirus, Aquamavirus, Avihepatovirus, Cardiovirus, Cosavirus, Dicipivirus, Enterovirus, Erbovirus, Hepatovirus, Kobuvirus, Megrivirus, Parechovirus, Salivirus, Sapelovirus, Senecavirus, Teschovirus, and Tremovirus [1]. To date, three species of kobuviruses have been identified, including Aichivirus A (formerly human Aichi virus), Aichivirus B (formerly bovine kobuvirus), and Aichivirus C (formerly porcine kobuvirus). Kobuviruses are new members of the Picornaviridae family. To date, kobuviruses have been detected in humans [2], cattle [3], pigs [4], sheep [5], bats [6], dogs [7], cats [8], ferrets [9], and goats [10]; this indicates that these viruses have a wide range of host species. The kobuviruses have the same genome organization, which includes a 5′UTR, a leader (L) protein, three structural proteins (VP0, VP3, and VP1), seven non-structural proteins (2A–2C and 3A–3D), and a 3′UTR.

Porcine kobuvirus was first detected in fecal samples from domestic pigs in Hungary, in 2007 [4]. Recently, porcine kobuviruses have also been detected at a high frequency in stool samples from healthy pigs in Hungary, China [11], Japan [12], Thailand [13], Brazil [14], The Netherlands [14], and Korea [15]. In addition, the prevalence of these viruses in pigs with diarrhea was shown to be very high in China during the last two years. Since December, 2010, there has been a large-scale outbreak of diarrheal disease in suckling piglets, characterized by watery diarrhea, dehydration, and vomiting. This outbreak led to 80% morbidity and 60% mortality in affected piglets in several farms in the Gansu Province of China. A recent report suggested that porcine kobuvirus infection correlated with diarrhea in pigs [15]. Porcine kobuviruses are a newly recognized viral agent in animals. Little is known about this virus, and only eight porcine kobuvirus genome sequences are available in GenBank. For this reason, it is important to increase the awareness of this disease and to stimulate further clinical, epidemiological, and molecular studies regarding porcine kobuviruses. In this study, we report four complete genome sequences and describe the detailed genomic organization of two porcine kobuvirus variant strains.

## 2. Results and Discussion

### 2.1. Prevalence and Complete Genomic Sequences of Porcine Kobuviruses

Overall, five (31.3%) of 17 fecal samples and two (33.3%) of six serum samples tested positive for kobuvirus infection using Kv primers. A total of 11 overlapping cDNA clones spanning the entire genome of K-11/2012/CH, K-4/2012/CH, GS-1/2012/CH, and GS-2/2012/CH were obtained, and their nucleotide sequences have been deposited into the GenBank database. Their accession numbers are KC414936, KC424638, KC424639, and KC424649, respectively. The complete RNA genomes of the K-11/2012/CH and K-4/2012/CH strains were similar to the sequence of the Hungarian strain, which consists of 8210 nucleotides (nt), excluding the poly (A) tail. A large open reading frame (ORF) of 7467 nt encoding a potential polyprotein precursor of 2488 amino acids (aa) was found. This ORF was located at the 5′ end and was preceded by 576 nt; 167 nt followed the ORF prior to the poly (A) tail. Relative to the genomes of the traditional strains, the genomes of the GS-1/2012/CH and GS-2/2012/CH variant strains contained 8121 nt, excluding the poly (A) tail; a 90-nucleotide deletion in the 2B protein and a single nucleotide insertion in the 3′UTR were also observed (Table 1).

**Table 1:** Comparison of the nucleotide and amino acid sequences of the study strains to the sequences of S-1-HUN.

Region	Length (nt)	Nucleotide identity (%)				Amino acid identity (%)				Predicted N-terminal cleavage site		
		K-11	K-4	GS-1	GS-2	K-11	K-4	GS-1	GS-2	K1-1/K-4	GS-1/GS-2	S-1-HUN
5′UTR	576	97	95	95	95	-	-	-	-	-	-	-
L	585	87	87	86	86	93	93	93	93	-	-	-
VP0	1,098	83	87	87	85	90	96	96	93	Q/G	Q/G	Q/G
VP3	669	87	87	86	77	93	97	95	97	Q/H	Q/H	Q/H
VP1	762	85	86	82	85	96	96	89	96	Q/A	Q/A	Q/A
2A	408	89	88	88	87	95	93	90	93	Q/C	Q/C	Q/C
2B	585/495 *	87	87	76	77	97	93	95	96	Q/G	Q/G	Q/G
2C	1,005	92	90	91	91	98	98	98	98	Q/G	Q/G	Q/G
3A	270	87	87	87	88	91	93	93	93	Q/G	Q/G	Q/G
3B	102	96	89	92	91	100	97	100	100	Q/G	Q/G	Q/G
3C	576	86	88	88	88	95	99	97	98	Q/S	Q/C	Q/S
3D	1,407	93	93	93	93	98	98	99	99	Q/S	Q/S	Q/S
3′UTR	167/168 *	98	97	96	96	-	-	-	-	-	-	-
Structural (VP0–VP3)	2,329	81	86	85	83	93	97	94	95	-	-	-
Non-structural(2A–3D)	353/4,263 *	91	90	89	89	97	97	95	95	-	-	-
Complete genome	8,210/8,121	86	89	88	87	95	96	94	94	-	-	-

* Indicates the nucleotide length of the variant strains.

### 2.2. Analysis of the UTRs and Coding Regions of the Four Strains

An alignment of the sequences of the four genomic sequences with porcine kobuvirus strain S-1-HUN, revealed that the genome sequences of K-4/2012/CH, K-11/2012/CH, GS-1/2012/CH, and GS-2/2012/CH shared 89%, 86%, 88%, and 87% identity to the S-1-HUN at the nucleotide level, respectively (Table 1). The highest nucleotide identities (96% and 98%) between the variant strains and the S-1-HUN strain were observed in the 3′UTR (Table 1), and the lowest nucleotide identities (76% and 87%) between the variant strains and the S-1-HUN strain were observed in the 2B coding region (Table 1). K-4/2012/CH and K-11/2012/CH polyprotein sequences were analyzed for potential cleavage sites, and the sites were identical to those in theS-1-HUN strain (Table 1). However, the potential 3C/3D cleavage site for the GS-1/2012/CH and GS-2/2012/CH variant strains was Q/C, which differed from the Q/S sequence in the traditional porcine kobuvirus strains.

### 2.3. Recombination Analysis of the Variant Stains

**Figure 1:**
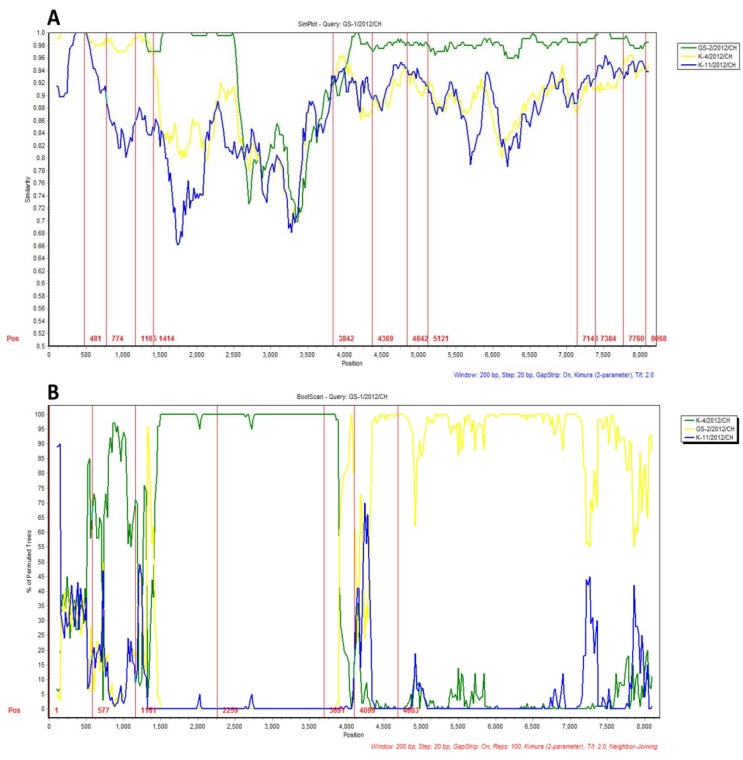
Nucleotide similarities and recombination among the kobuvirus genomes. (**A**) Nucleotide similarities among theK-4/2012/CH, K-11/2012/CH, GS-1/2012/CH, and GS-2/2012/CH genomes, as determined using the SimPlot program. The x-axis indicates the nucleotide position along the alignment, and the y-axis shows the similarity; (**B**) Bootscan plot of the GS-1/2012/CH strain with GS-1/2012/CH, K-4/2012/CH, and K-4/2012/CH reference sequences as out groups. The analysis was performed using pairwise distance, modeled with a window size of 200, step size of 20, and 100 bootstrap replicates.

The four complete kobuvirus genome sequences were analyzed using Simplot. The similarity plot displays the consecutive nucleotide identity (%) among the queried strain and parental strains. The Bootscan plots display the likelihood of recombination within the GS-1/2012/CH variant strain; the 481–1,414 and 3,482–4,100 nucleotide regions are similar to regions found in the K-4/2012/CH strain. The other genomic regions of the GS-1/2012/CH variant strain, including 4,100–4,369, 7,143–7,384, and 7,760–8,068, are closely related to the K-11/2012/CH strain (Figure 1A). Significant recombination signals were identified in the 481–774, 1,165–1,414, 3,842–4,369, 4,842–5,121, 7,143–7,384, and 7,760–8,068 nucleotide regions (Figure 1B).

### 2.4. Analysis of Coding Region Amino Acid Sequences

**Figure 2:**
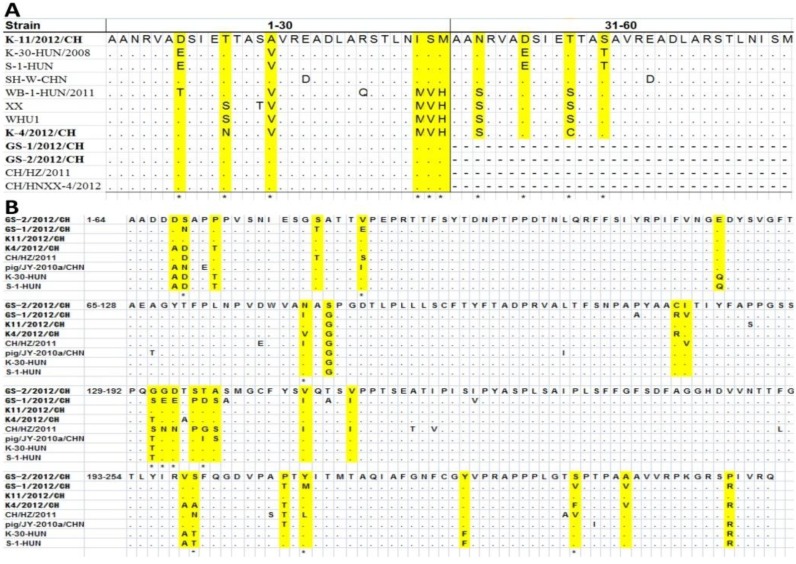
Comparison of the deduced protein amino acid sequences of the study strains and the reference strains. (**A**) Comparison of the deduced 2B protein amino acid sequences. An asterisk (*) indicates an amino acid substitution in the VP1 region; a dash (-) indicates an amino acid deletion in the variant strain; (**B**) Comparison of the deduced VP1 amino acid sequences. An asterisk (*) indicates no fewer than three amino acid substitutions within the VP1 region.

The structural and non-structural protein regions of the both K-4/2012/CH and K-11/2012/CH strains contain 2329aa and 4353aa, respectively. The amino acid identity identities among the of structural regions (VP0–VP3) are 90%–97%, and the non-structural regions (2A–3D) share 93%–100% amino acid identity with that of the S-1-HUN strain (Table 2). Compared with the K-4/2012/CH and K-11/2012/CH strains, the variant strain has a 30-animo acid deletion within the 2B protein. The amino acid identities among the structural regions (VP0–VP3) are 89%–97%, and the non-structural regions (2A–3D) share 90%–100% amino acid identity with that of the S-1-HUN strain (Table 2). The 2B amino acid sequence alignment for the 12 strains analyzed in this study and the eight strains from the GenBank database (K-30-HUN/2008 and S-1-HUN are Hungarian strains; others are Chinese strains) is shown in Figure 2A. Ten amino acid mutations were observed at positions 7, 11, 15, 28, 29, 30, 33, 37, 41, and 44. Though the function of the porcine kobuvirus 2B protein is not known, protein 2B has been shown to assume different functions in different kobuviruses [15].

**Table 2:** Kobuvirus strains used in this study.

Accession No.	Virus strain	Kobuvirus species	Host species	Country reported
GQ927711	D/VI2287/2004	Aichi virus	Human	Germany
JX564249	kvgh99012632/2010	Aichi virus	Human	Taiwan
DQ028632	Goiania/GO/03/01/Brazil	Aichi virus	Human	Brazil
FJ890523	Chshc7	Aichi virus	Human	China
AB040749	A846/88	Aichi virus	Human	Japan
HQ650197	KB101	Bovine virus	Cattle	South Korea
AB084788	U-1	Bovine virus	Cattle	Japan
HQ650168	KB12	Bovine virus	Cattle	South Korea
HQ650180	KB15	Bovine virus	Cattle	South Korea
JF755427	M-5/USA/2010	Mouse virus	Mouse	USA
JN387133	AN211D/USA/2009	Dog virus	Dog	USA
KF006985	MpKoV38	Ferret virus	Ferret	Netherlands
GU245693	TB3/HUN/2009	Sheep virus	Sheep	Hungary
HQ400969	97DA4	Porcine virus	*Sus scrofa*	South Korea
HQ400970	111DA18	Porcine virus	*Sus scrofa*	South Korea
HQ400968	99DA6	Porcine virus	*Sus scrofa*	South Korea
HQ400967	110DA17	Porcine virus	*Sus scrofa*	South Korea
HQ400963	95DA2	Porcine virus	*Sus scrofa*	South Korea
HQ400966	123DB10	Porcine virus	*Sus scrofa*	South Korea
HQ400962	94DA1	Porcine virus	*Sus scrofa*	South Korea
HQ400948	15OA16	Porcine virus	*Sus scrofa*	South Korea
JF422792	D11	Porcine virus	Pig	China
JF422788	D1	Porcine virus	Pig	China
JX401523	CH/HNXX-4/2012	Porcine virus	Piglet	China
NC_016769	SH-W-CHN	Porcine virus	Pig	China
JX177612	WB-1-HUN/2011	Porcine virus	*Sus scrofa*	Hungary
JQ692069	WHU1	Porcine virus	*Sus scrofa*	China
GQ249161	K-30-HUN/2008	Porcine virus	*Sus scrofa*	Hungary
EU787450	S-1-HUN	Porcine virus	*Sus scrofa*	Hungary
JX827598	CH/HZ/2011	Porcine virus	Pig	China
GU298971	Ch16/2008/CHN	Porcine virus	Pig	China
GU298967	Ch40/2008/CHN	Porcine virus	Pig	China
GU292559	JY-2010a/CHN	Porcine virus	Pig	China
KC414936	K-11/2012/CH	Porcine virus	*Sus scrofa*	China
KC424638	K-4/2012/CH	Porcine virus	*Sus scrofa*	China
KC424639	GS-1/2012/CH	Porcine virus	*Sus scrofa*	China
KC424649	GS-2/2012/CH	Porcine virus	*Sus scrofa*	China
KC204684	XX	Porcine virus	Pig	China

VP1 is highly exposed and is the most immunodominant part of the kobuvirus capsid protein [3], and this protein is the most variable among the structural proteins [16]. Eight VP1 regions were analyzed in this study, and more than three amino acid mutations were found specifically at positions 6 (S, N, and D); 22 (E, S, and I); 81 (N, I, and V); 131 (G, S, and T); 132 (G, E, and N); 133 (D, E, and N); 136 (T, D, G, and I); 199 (S, A, N, and T); 209 (Y, M, and L); and 235 (S, V, and F) (Figure 2B).

### 2.5. Phylogenetic Analysis

**Figure 3:**
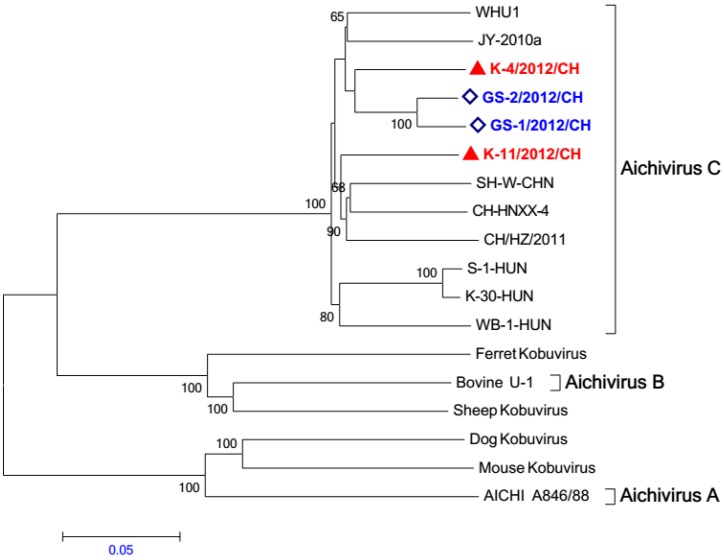
Relationships among kobuviruses, including porcine kobuvirus and other kobuviruses (human, bovine, sheep, dog, ferret, and mouse) based on whole genome sequences. Bootstrap values (based on 1,000 replicates) for each node are provided when >50%.

We compared the entire genome sequences of K-4/2012/CH, K-11/2012/CH, GS-1/2012/CH, and GS-2/2012/CH with those of other kobuviruses. A phylogenetic tree based on whole genome sequences indicted that all four of the study sequences were placed in the porcine kobuvirus genetic clade, which was separate from the other kobuviruses (Figure 3). Within this clade, the K-4/2012/CH, K-11/2012/CH, GS-1/2012/CH, and GS-2/2012/CH strains form two groups; the GS-1/2012/CH and GS-2/2012/CH variant strains form a separate group with the other porcine kobuviruses.

**Figure 4:**
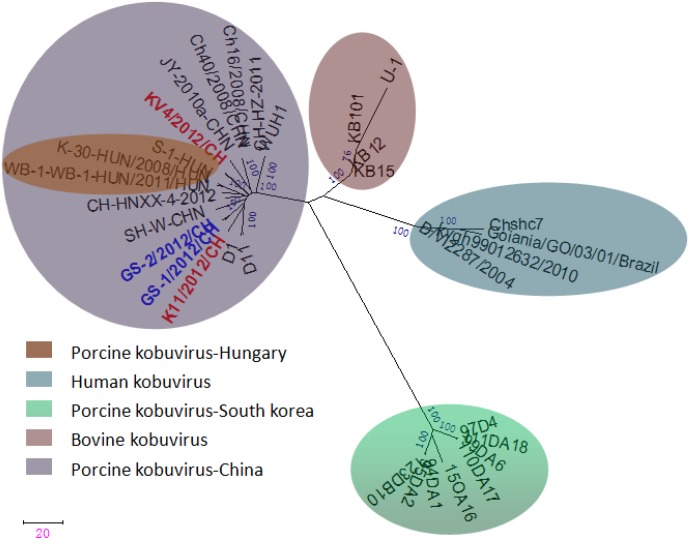
Phylogenetic tree of porcine kobuviruses based on the sequences from the 3D region (588 nt). The phylogenetic tree was constructed using the neighbor-joining clustering method; distance was calculated using the maximum composite likelihood correction for evolutionary rate in MEGA version 5.0. Bootstrap values (based on 1,000 replicates) for each node are provided when >50%.

The sequences of the 3D region have been particularly useful for placing viruses within species or genera and for comparing viruses of different genera or families [17]. A phylogenetic analysis shows two genetic lineages of porcine kobuviruses, however, these viruses are phylogenetically distinct from both bovine kobuviruses and Aichi viruses based on partial sequences of the 3D region (Figure 4). The four porcine kobuviruses isolated in our study and the Hungarian strains were in the same lineage, and the South Korean strains were placed in another lineage. 

### 2.6. Viral Culture

No cytopathic effects (CPE) were observed in Vero, BHK-21 (Baby Hamster Kidney), or PK-15 (Pig Kidney) cells following serial passage of cultures. Porcine kobuvirus RNA replication were not detected in the cells and supernatants in every passage were collected. 

## 3. Experimental

### 3.1. Fecal and Serum Samples

Seventeen fecal and serum samples were collected in July, 2012, from dead piglets; these piglets showed signs of watery diarrhea and dehydration for <10 days and were from two farms (located in Jiayuguan and Linxia City) in Gansu Province. Six serum samples from a one-year-old pig in Jiuquan City within Gansu Province were also obtained. Fecal samples were prepared as 10% homogenates in PBS and then centrifuged at 10,000 ×*g* for 20 min. The resultant supernatants were applied to Vero, PK, or BHK cells prior to RT-PCR analysis. 

### 3.2. RT-PCR, Cloning, and Sequencing

Total RNA was extracted from fecal and serum samples using TRIzol reagent according to the manufacturer’s instructions (Invitrogen Life Technologies, Grand Island, NY, USA). RNA was reverse transcribed with avian myeloblastosis virus reverse transcriptase (Promega, Beijing, China) in the presence of random primersand oligo dT15 (TaKaRa Bio Inc., Otsu, Japan). The cDNA was immediately amplified with primers that were designed based on the sequences of kobuvirus reference strains (Table 3). The 5′ and 3′ ends of the genome were confirmed using a SMARTer rapid amplification of cDNA ends (RACE) cDNA amplification kit (Clontech, Japan). PCR reactions contained 1 µL of FastPfu DNA Polymerase (TransGen Biotech, Beijing, China), 10 µL of 5× FastPfu Buffer, 5 µL of dNTP mixture (2.5 mM), 1 µL of each primer, 2 µL of template, and sterile H_2_O to yield a final volume of 50 µL. The PCR conditions were as follows: 94 °C for 5 min; 35 cycles of 94 °C for 30 s, 55 °C for 30 s, and 72 °C for 1 min; and a final extension at 72 °C for 10 min. Amplicons were separated in a 1.5% agarosegel.

**Table 3:** Primers designed from contigs to acquire the porcine kobuvirus genomes.

Primer name	Sequence (5'–3')	Size of PCR product (bp)
Pokv-30F	CCCTCACCCTCTTTTCCG	369
Pokv-398R	ACCGCAGTCCATGCTCTA	
Pokv-302F	AAACTCCTACCCGACAAA	966
Pokv-1267R	CAGGRCCATCACCAAGRC	
Pokv-1134F	YAMCACTCCTTGCCAGATC	1048
Pokv-2181R	AGAACCAGTWGGRACAGM	
Pokv-2048F	ACCTCTAYCAGGGCAAYA	955
Pokv-3002R	GGTTCAGGGACWGTAGTAGC	
Pokv-2894F	TSCGYGGCATCCAAGCAC	946
Pokv-3839R	AGACCAAGGCGGGAAAGG	
Pokv-3723F	CTGTCCAGACGTGCGGRTYT	1019
Pokv-4741R	CCATGAGCCACTCGGTGTTC	
Pokv-4668F	GACGGTTGAACAYCAAGGTG	928
Pokv-5595R	GAARGAAGGYTGCCAAAGAG	
Pokv-5462F	ARCCCTTCGAYCCTGTGGAG	925
Pokv-6386R	GGAGGACCAGAAAGAGTAGAAAT	
Pokv-6219F	YATCGGTCCRGACACCTTTG	970
Pokv-7188R	GATAGCGTGKAYGGGAGCAG	
Pokv-7036F	TTGCCRMYTCCWGAGTTRGA	695
Pokv-7730R	AAAGTRTCTGTTTTRTTTGCTG	
Pokv-7614F	CTAYGGTGATGATGTGATCTATG	582
Pokv-8195R	AAGTAAAGGACAGCCAGGGA	
Kv-F	TCTGGATGCGTTGGCACTTCCAT	519
Kv-R	CCAGCGGGTCTGAAGGTAAGAGT	

### 3.3. Sequencing and Phylogenetic Analysis

PCR products were cloned into the pMD-18 T vector (TaKaRa, Otsu, Japan). Competent *E. coli* DH5a cells (TaKaRa) were transformed with the recombinant plasmid. Three clones were sequenced using the Applied Biosystems (ABI) 3730xl DNA analyzer. Sequences were analyzed using the ClustalX version 2.0 [18], DNASTAR, and MegAlign version 5.0 (DNAStar Inc., Madison, WI, USA) software packages. A dendrogram was constructed using the neighbor-joining clustering method in MEGA 5.0 [18]. Possible recombination events in the coding region were analyzed using Simplot software [20]. All kobuvirus strains used in the study are listed in Table 2. 

### 3.4. Viral Culture

Original fecal samples that tested positive for porcine kobuvirus by RT-PCR were propagated separately for virus isolation. Vero, PK-15, and BHK-21 cells were used as general-purpose cell lines for propagating picornaviruses.

## 4. Conclusions

The first porcine kobuvirus was identified in Hungary in 2007. Since then, porcine kobuviruses have been reported in several Asian countries. Porcine kobuviruses are not geographically restricted and are widely distributed in pigs worldwide. Porcine kobuviruses were also detected at a high frequency in pigs without clinical diarrhea [16]. Here, we report the complete nucleotide sequences and genetic organization of two traditional strains (K-4/2012/CH and K-11/2012/CH) and two variant strains (GS-1/2012/CH and GS-2/2012/CH).

The complete genome sequences of four strains were compared to the prototype porcine kobuvirus strain (S-1-HUN). The genomes of the K-11/2012/CH and K-4/2012/CH strains were similar to that of the Hungarian strain, which consists of 8210 nucleotides. However, the variant strains, GS-1/2012/CH and GS-2/2012/CH, contain 8121 nucleotides, with a 90-nucleotide deletion within the 2B protein and a single nucleotide insertion within the 3′UTR. Though the function of the porcine kobuvirus 2B protein is unknown, the 2B protein has many different functions in different kobuviruses [21]. In our study, ten amino acid mutations were observed in the 2B protein-coding region, though further studies are required to address whether the variant strains cause serious diarrheal outbreaks in pigs.

The VP1 regions of the four porcine kobuviruses in our study were highly variable [22]. The overall nucleotide identity among the VP1 sequences was 81%–86% (Table 1). Ten amino acid mutations were consistently observed at certain positions within the VP1 region. The mechanisms responsible for the evolution of the viral RNA sequences remain largely unknown. Host, viral, and environmental factors may play roles in this process [23].

Natural recombination has been reported in most picornavirus genera, and it is a key genetic feature of these infectious agents [24]. By exchanging sequences, the virus may induce changes in its genome sequence. This may lead to the acquisition of many key adaptive mutations [25], thus generating genetic variation and even producing new viral variants. One striking difference between porcine kobuvirus and the members of the other two kobuvirus species is that the 5′UTR of porcine kobuvirus contains an IRES related to that of hepatitis C virus [26,27] , whereas the 5′UTRs of the other species contain IRESs that are structurally and mechanistically unrelated [28,29]. In our study, significant recombination signals were identified using SimPlot scans of whole genome sequences. However, kobuvirus recombination cannot be discussed without also considering other evolutionary properties, such as global prevalence and its dynamic epidemiology.

Kobuviruses are transmitted primarily by the fecal-oral route, and other picornaviruses may use a similar mechanism [16]. Porcine kobuviruses are thought to be confined to the intestine, but porcine kobuvirus viremia has also been recently reported [5]. We also detected porcine kobuvirus sequences in pig serum. This finding suggests that porcine kobuvirus can escape the gastrointestinal tract and travel to the circulatory system. In addition to fecal-oral transmission, further experiments are necessary to investigate the possibility of kobuvirus transmission through breastfeeding and via blood-borne infections.

In summary, porcine kobuviruses are a newly recognized viral agent in animals. Porcine kobuviruses maybe persist for long periods because this viral infection can be asymptomatic. The presence of mutations and recombination events may contribute to the high levels of genetic diversity in porcine kobuviruses. The genomic changes and viremia may make this virus a potentially dangerous disease-causing agent in general, particularly in humans. 

## References

[1] International Committee on Taxonomy of Viruses ICTV official taxonomy: Updates since the 9th report. http://talk.ictvonline.org/files/ictv_documents/m/msl/4440.aspx.

[2] Yamashita T., Kobayashi S., Sakae K., Nakata S., Chiba S., Ishihara Y., Isomura S. (1991). Isolation of cytopathic small round viruses with BS-C-1 cells from patients with gastroenteritis. J. Infect. Dis..

[3] Yamashita T., Ito M., Kabashima Y., Tsuzuki H., Fujiura A., Sakae K. (2003). Isolation and characterization of a new species of kobuvirus associated with cattle. J. Gen. Virol..

[4] Reuter G., Boldizsar A., Kiss I., Pankovics P. (2008). Candidate new species of Kobuvirus in porcine hosts. Emerging Infect. Dis..

[5] Reuter G., Kecskemeti S., Pankovics P. (2010). Evolution of porcine kobuvirus infection, Hungary. Emerging Infect. Dis..

[6] Li L., Victoria J.G., Wang C., Jones M., Fellers G.M., Kunz T.H., Delwart E. (2010). Bat guano virome: Predominance of dietary viruses from insects and plants plus novel mammalian viruses. J. Virol..

[7] Li L., Pesavento P.A., Shan T., Leutenegger C.M., Wang C., Delwart E. (2011). Viruses in diarrhoeic dogs include novel kobuviruses and sapoviruses. J. Gen. Virol..

[8] Chung J.Y., Kim S.H., Kim Y.H., Lee M.H., Lee K.K., Oem J.K. (2013). Detection and genetic characterization of feline kobuviruses. Virus Genes.

[9] Smits S.L., Raj V.S., Oduber M.D., Schapendonk C.M., Bodewes R., Provacia L., Stittelaar K.J., Osterhaus A.D., Haagmans B.L. (2013). Metagenomic analysis of the ferret fecal viral flora. PLoS One.

[10] Lee M.H., Jeoung H.Y., Lim J.A., Song J.Y., Song D.S., An D.J. (2012). Kobuvirus in South Korean black goats. Virus Genes.

[11] Yu J.M., Jin M., Zhang Q., Li H.Y., Li D.D., Xu Z.Q., Li J.S., Cui S.X., Yang S.H., Liu N. (2009). Candidate porcine Kobuvirus, China. Emerging Infect. Dis..

[12] Khamrin P., Maneekarn N., Hidaka S., Kishikawa S., Ushijima K., Okitsu S., Ushijima H. (2010). Molecular detection of kobuvirus sequences in stool samples collected from healthy pigs in Japan. Infect. Genet. Evol..

[13] Khamrin P., Maneekarn N., Kongkaew A., Kongkaew S., Okitsu S., Ushijima H. (2009). Porcine kobuvirus in piglets, Thailand. Emerging Infect. Dis..

[14] Barry A.F., Ribeiro J., Alfieri A.F., van der Poel W.H., Alfieri A.A. (2011). First detection of kobuvirus in farm animals in Brazil and The Netherlands. Infect. Genet. Evol..

[15] Park S.J., Kim H.K., Moon H.J., Song D.S., Rho S.M., Han J.Y., Nguyen V.G., Park B.K. (2010). Molecular detection of porcine kobuviruses in pigs in Korea and their association with diarrhea. Arch. Virol..

[16] Reuter G., Boros A., Pankovics P. (2011). Kobuviruses—A comprehensive review. Rev. Med. Virol..

[17] Oberste M.S., Maher K., Pallansch M.A. (2003). Genomic evidence that simian virus 2 and six other simian picornaviruses represent a new genus in Picornaviridae. Virology.

[18] Larkin E.K., Morris N.J., Li Y., Nock N.L., Stein C.M. (2007). Comparison of affected sibling-pair linkage methods to identify gene x gene interaction in GAW15 simulated data. BMC Proc..

[19] Tamura K., Peterson D., Peterson N., Stecher G., Nei M., Kumar S. (2011). MEGA5: Molecular evolutionary genetics analysis using maximum likelihood, evolutionary distance, and maximum parsimony methods. Mol. Biol. Evol..

[20] Lole K.S., Bollinger R.C., Paranjape R.S., Gadkari D., Kulkarni S.S., Novak N.G., Ingersoll R., Sheppard H.W., Ray S.C. (1999). Full-length human immunodeficiency virus type 1 genomes from subtype C-infected seroconverters in India, with evidence of intersubtype recombination. J. Virol..

[21] De Jong A.S., de Mattia F., van Dommelen M.M., Lanke K., Melchers W.J., Willems P.H., van Kuppeveld F.J. (2008). Functional analysis of picornavirus 2B proteins: Effects on calcium homeostasis and intracellular protein trafficking. J. Virol..

[22] Okitsu S., Khamrin P., Thongprachum A., Hidaka S., Kongkaew S., Kongkaew A., Maneekarn N., Mizuguchi M., Hayakawa S., Ushijima H. (2012). Sequence analysis of porcine kobuvirus VP1 region detected in pigs in Japan and Thailand. Virus Genes.

[23] Wang C., Lan D., Cui L., Yang Z., Yuan C., Hua X. (2012). Molecular characterization of a porcine kobuvirus strain in China. Arch. Virol..

[24] Lukashev A.N. (2010). Recombination among picornaviruses. Rev. Med. Virol..

[25] Kuiken T., Holmes E.C., McCauley J., Rimmelzwaan G.F., Williams C.S., Grenfell B.T. (2006). Host species barriers to influenza virus infections. Science.

[26] Hellen C.U., de Breyne S. (2007). A distinct group of hepacivirus/pestivirus-like internal ribosomal entry sites in members of diverse picornavirus genera: Evidence for modular exchange of functional noncoding RNA elements by recombination. J. Virol..

[27] Reuter G., Boldizsar A., Pankovics P. (2009). Complete nucleotide and amino acid sequences and genetic organization of porcine kobuvirus, a member of a new species in the genus *Kobuvirus*, family Picornavirida. Arch. Virol..

[28] Yu Y., Sweeney T.R., Kafasla P., Jackson R.J., Pestova T.V., Hellen C.U. (2011). The mechanism of translation initiation on Aichivirus RNA mediated by a novel type of picornavirus IRES. EMBO J..

[29] Sweeney T.R., Dhote V., Yu Y., Hellen C.U. (2012). A distinct class of internal ribosomal entry site in members of the Kobuvirus and proposed Salivirus and Paraturdivirus genera of the Picornaviridae. J. Virol..

